# Comparison and Identification of Estrogen-Receptor Related Gene Expression Profiles in Breast Cancer of Different Ethnic Origins

**DOI:** 10.4137/bcbcr.s626

**Published:** 2008-03-25

**Authors:** Hsiao-Wei Chen, Hsuan-Cheng Huang, Yi-Shing Lin, King-Jen Chang, Wen-Hung Kuo, Hsiao-Lin Hwa, Fon-Jou Hsieh, Hsueh-Fen Juan

**Affiliations:** 1 Department of Life Science, National Taiwan University, Taipei 106, Taiwan; 2 Institute of Biomedical Engineering, National Taiwan University, Taipei 106, Taiwan; 3 Institute of Biomedical Informatics, National Yang-Ming University, Taipei 112, Taiwan; 4 Welgene Biotech. Co., Ltd., Taipei, Taiwan; 5 Department of Surgery, College of Medicine, National Taiwan University, Taipei 106, Taiwan; 6 Obstetrics and Gynecology, College of Medicine, National Taiwan University, Taipei 106, Taiwan; 7 Institute of Molecular and Cellular Biology, National Taiwan University, Taipei 106, Taiwan; 8 Institute of Biomedical Electronics and Bioinformatics, National Taiwan University, Taipei 106, Taiwan; 9 Center for Systems Biology and Bioinformatics, National Taiwan University, Taipei 106, Taiwan

**Keywords:** estrogen receptor, breast cancer, microarray, gene expression profile

## Abstract

The interactions between genetic variants in estrogen receptor (ER) have been identified to be associated with an increased risk of breast cancer. Available evidence indicates that genetic variance within a population plays a crucial role in the occurrence of breast cancer. Thus, the comparison and identification of ER-related gene expression profiles in breast cancer of different ethnic origins could be useful for the development of genetic variant cancer therapy. In this study, we performed microarray experiment to measure the gene expression profiles of 59 Taiwanese breast cancer patients; and through comparative bioinformatics analysis against published U.K. datasets, we revealed estrogen-receptor (ER) related gene expression between Taiwanese and British patients. In addition, SNP databases and statistical analysis were used to elucidate the SNPs associated with ER status. Our microarray results indicate that the expression pattern of the 65 genes in ER+ patients was dissimilar from that of the ER- patients. Seventeen mutually exclusive genes in ER-related breast cancer of the two populations with more than one statistically significant SNP in genotype and allele frequency were identified. These 17 genes and their related SNPs may be important in population-specific ER regulation of breast cancer. This study provides a global and feasible approach to study population-unique SNPs in breast cancer of different ethnic origins.

## Introduction

Breast cancer is one of the most common cancers for women in the world as it ranks number one among other cancers in developed countries, and ranks fourth in Taiwan. Current research suggests that interactions between genetic variants and a wide range of environmental factors may contribute to the development of breast cancer. Available evidence indicates that genetic variance within the population plays a role in the probability of breast cancer development, with a low incidence in certain groups of Asian women to the highest in Caucasian women (Hsiao et al. 2004).

With microarray technique, large amounts of gene expression data can be obtained in a short period of time. Gene expression profiling is a powerful tool for identifying gene activity patterns, which enables the distinction among various subtypes of breast cancer (including luminal subtypes A and B, and ERBB2 between basal and normal) (Sorlie et al. 2001). According to the data from both clinical and animal studies, estrogen is crucial to the development and progression of breast cancer. Estrogen mediates its effects through the estrogen receptor (ER), which serves as the basis for many therapeutic interventions (Deroo and Korach, 2006). More than two-thirds of breast cancers show estrogen receptor expression at the time of diagnosis, and immunohistochemical detection of estrogen receptor expression is routinely used in making decisions on hormonal therapy for breast cancer (Holst et al. 2007). Gene variants in steroid hormone related genes, ESR1, ESR2, PGR, and HSD17B1 have been identified to be associated with either an increased or decreased risk of breast cancer (Feigelson et al. 2006; Gold et al. 2004); however, the exact associations remain unclear. Furthermore, ER-α allelic variants have been reported to be associated with the risk for breast cancer (Gold et al. 2004) in Caucasians and in Taiwanese (Hsiao et al. 2004). Certain single nucleotide polymorphisms (SNPs) may influence the regulation of ERs and coregulators on tumor development and progression. In this study, we developed an approach to find out population-unique SNPs in breast cancer of different ethnic origins by comparing the gene expression profiles of two different populations and related SNP data.

## Materials and Methods

### Tumor tissue samples and examination of ER

Surgical specimens of breast cancer tumor tissue were freshly collected and snap frozen from patients who underwent surgery at National Taiwan University Hospital (NTUH) between 2002 and 2005. Cancer samples containing relatively pure tumor, as defined by greater than 50% tumor cells per high-power field examined in a section adjacent to the tissue used, were included in this study. All the paraffin sections of breast cancer specimens (3–5 m in thickness) on slides were processed in Ventana’s automated staining system (BenchMark â LT) (Ventana Medical System Inc., Tucson, AZ, U.S.A.) for the immunohistochemical stain (IHC). Firstly the slides were probed with CONFIRMTM anti-Estrogen Receptor (SP1) rabbit monoclonal primary antibody (Catalog # 790–4325, Ventana Medical System Inc.). Secondly, to localize and visualize ER protein within the specimen, iVIEW TM DAB Detection kit (Catalog # 760-091, Ventana Medical System Inc.) was applied. The negative control slides for tumor specimens were solely stained using iVIEWTM DAB Detection kit (Catalog # 760-091, Ventana Medical System Inc.).

### RNA extraction and oligo microarray

Total RNA was extracted by Trizol^®^ Reagent (Invitrogen, U.S.A.), followed by RNeasy Mini Kit (Qiagen, Germany). Purified RNA is quantified at OD260nm by a ND-1000 spectrophotometer (Nanodrop Technology, U.S.A.) and quality-controlled by Bioanalyzer 2100 (Agilent Technology, U.S.A.). A human reference RNA pooled from 10 cell lines (Stratagene, U.S.A.) was used to serve as reference in microarray comparison. 0.5 g of total RNA was amplified by a Low RNA Input Fluor Linear Amp kit (Agilent Technologies) and labeled with Cy3 or Cy5 (CyDye, PerkinElmer, U.S.A.) during the *in vitro* transcription process. Tumor RNA was labeled with Cy5 and RNA from Universal Human Reference RNA was labeled with Cy3. 2 g of Cy-labeled cRNA was fragmented to an average size of about 50–100 nucleotides by incubating with fragmentation buffer at 60 °C for 30 minutes. Correspondingly fragmented labeled cRNA is then pooled and hybridized to Human 1A (version 2) oligo microarray (Agilent Technologies) at 60 °C for 17 h. After washing and drying with nitrogen gun blowing, microarrays are scanned with an Agilent microarray scanner (Agilent Technologies, U.S.A.) at 535 nm for Cy3 and 625 nm for Cy5. Scanned images are analyzed by Feature Extraction software 6.0 (Agilent Technologies, U.S.A.), and each feature is quantified by Feature Extraction to output the signal and background intensity; the data are substantially normalized by rank-consistency-filtering LOWESS method.

### Microarray data analysis

**Figure 1.**
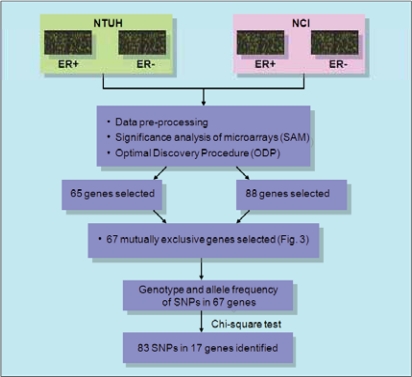
**Flowchart for the gene expression-based comparative analysis.** Microarray gene expression data of the NCI dataset (breast cancer patients in the U.K.) and the NTUH dataset (breast cancer patients in Taiwan) were separated into ER+ and ER− groups respectively for further identification of significantly differentially expressed genes through statistical algorithms, Significant Analysis of Microarrays (SAM) and Optimal Discovery Procedure (ODP). 88 genes in the NCI dataset and 65 genes in the NTUH dataset were selected. After mapping these selected genes against the common gene pool between both datasets, 67 mutually exclusive genes were chosen for the SNP analysis. After examining the genotype and allele frequency of these 67 genes using chi-square test (p-value <0.001), 83 SNPs in the 17 genes were identified.

In this study, we used microarray technique to profile the gene expression of 59 breast cancer patients in Taiwan with primary invasive breast carcinoma. For the comparison of the gene expression profiles between Taiwanese and U.K. patients, we used U.K. breast cancer data from the National Cancer Institute (NCI) which were obtained from the online supplementary materials of Sotiriou et al. (Sotiriou et al. 2003). Detailed information and clinical characteristics for breast cancer patients in the U.K. and Taiwan are shown in Table 1. The gene expression datasets from our microarray results and that of NCI were grouped into ER+ and ER− respectively according to their clinical prognosis variables shown in Figure 1. To identify differentially expressed genes between the two groups and to increase the accuracy of significant gene selection, combination of Significance Analysis of Microarrays (SAM) (Tusher et al. 2001) and Optimal Discovery Procedure (ODP) (Storey, 2005) were jointly used for the selection of differentially expressed genes.

### Hierarchical clustering

Hierarchical clustering was processed by Multiple-Experiment Viewer (MeV) 4.0, an open source software which is part of the TM4 Software Suite (Saeed et al. 2003) created by The Institute for Genomic Research (TIGR, Rockville, MD). Euclidean distance and average linkage were used to measure the distance of gene expression.

### SNP search and statistical analysis

SNP information was retrieved from the Perlegen Genotype Browser (http://genome.perlegen.com/browser/index_v2.html) and dbSNP (http://www.ncbi.nlm.nih.gov/projects/SNP/). Perlegen Genotype Browser and dbSNP contain SNP information of three ethnic populations, African American, European American, and Chinese. For the purpose of this study, we focused on the SNPs of European American and Chinese to represent the United Kingdom (U.K.) and Taiwanese populations respectively for further statistical analysis. SNPs in the 67 candidate genes were initially searched and browsed through the Perlegen Genotype Browser. The SNPs with differences in allele frequencies between European American and Chinese were manually screened for further analysis. In order to collect detailed information on the rest of the SNPs, we referred to dbSNP and retrieved the genotype and allele frequencies of the two populations. Through Pearson’s chi-square tests, the SNPs of our candidate genes that were significantly differentiated in both genotype and allele frequencies between European American and Chinese were identified. We utilized R software for the statistical analysis of SNPs.

## Results

### Differentially expressed genes in ER+/ER− breast cancer subgroups

**Figure 2.**
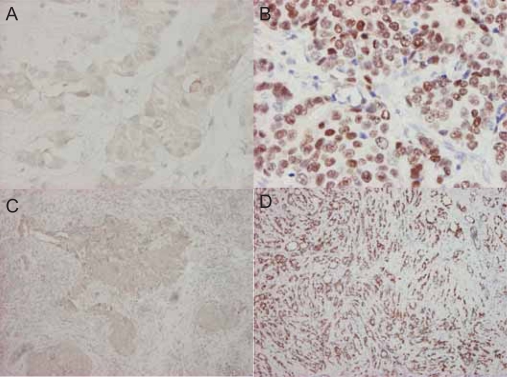
**Immunohistochemical stain of ER in Taiwanese breast cancer tissues.** (**A**) and (**C**) ER− 200X and 40X; (**B**) and (**D**) ER+ 200X and 40X. The microarray experiments were performed by using these examined tissues.

**Figure 3.**
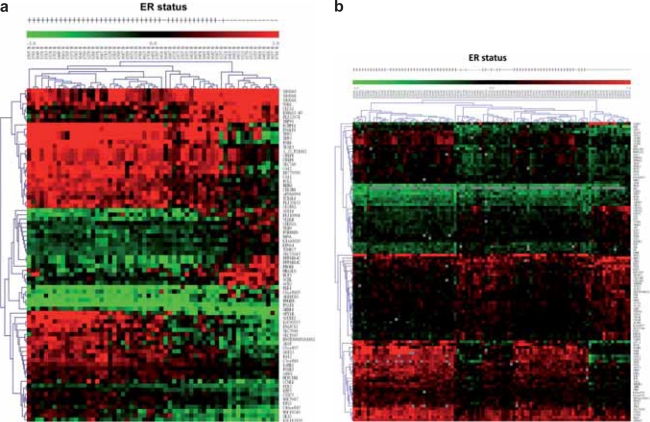
**Hierarchical clustering diagrams of (a) NTUH dataset and (b) NCI dataset.** Genes with similar expression pattern (vertical) and breast cancer samples with similar ER statuses (horizontal) were hierarchically clustered together. For the NTUH dataset, the majority of ER+ samples are on the left region (from 1595 M to 1257 M, except for 1431 M and 1587 M) while the majority of ER− are on the right; for the NCI dataset, few ER− samples fell under the ER+ group (X 21643 and X21618), as most ER− samples were clustered in the middle or on the right The clustering results confirmed our selection of differentially expressed genes in ER+/ER− groups, representing distinct gene expressions between NTUH and NCI datasets.

First, we used immunohistochemical stain to examine ER+/ER− breast cancer tissue subgroups (Fig. 2). The microarray data were grouped into ER+ and ER− groups based on the ER status of breast cancer patients. In order to select the differentially expressed genes, we combined two algorithms, Significance Analysis of Microarray (SAM) (Tusher et al. 2001) and Optimal Discovery Procedure (ODP) (Storey, 2005). Using two-class unpaired SAM supervised analysis, and setting the imputation engine at 10 *K*-nearest neighbors, we found 78 differentially expressed genes from our data, in which 31 of them were up-regulated and 47 were down-regulated. After applying ODP algorithm and imputing missing data, 737 genes with q-value under cut-off at 0.01 were then selected. 65 genes were simultaneously picked up by both SAM and ODP analysis, and were regarded as the most significant genes. The flowchart for candidate gene selection is shown in Figure 1. The log_2_ transformed data of the genes differentially expressed between ER+ and ER− groups are shown in the hierarchical clustering diagram (Fig. 3a). The expression pattern of the 65 genes in ER+ patients (Fig. 3a, left) was dissimilar from that of the ER− patients (Fig. 3a, right). Similarly, in the NCI dataset, 107 genes were selected using SAM algorithm (genes with missing gene symbol were excluded); 56 of them were up-regulated and 51 were down-regulated. 900 genes were considered as significant genes by the ODP algorithm, and these genes included the 88 genes selected by SAM, which were regarded as the most significant genes in the NCI dataset. Hierarchical clustering diagram displays the gene expression pattern of these 88 genes in Figure 3b.

### Comparison of differentially expressed genes in our and NCI data

**Figure 4.**
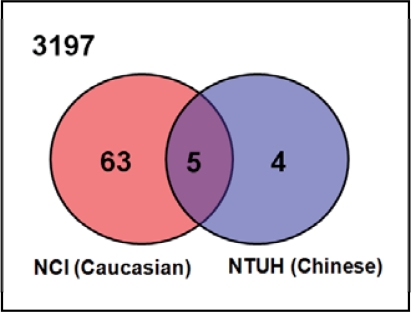
**The Venn diagram of significant genes in our and NCI datasets.** In order to compare the difference of differentially expressed genes selected in NTUH and NCI datasets, we only focus on the gene probes common in both datasets. After filtering out the genes only present in either NTUH or NCI datasets, a total number of 3197 genes were left, and were considered as our common candidate gene pool for further identification. In the NCI dataset, 68 of the 88 differentially expressed genes (refer to Fig. 1) were in the pool; while NTUH dataset has 9 genes left in the pool. The 67 mutually exclusive genes were selected for further SNP analysis.

The microarrays used in our and NCI datasets have 3197 gene probes in common, and were designated as our candidate gene pool for analysis. Shown in Figure 4, with regard to our candidate gene pool, in the NTUH dataset, 9 of 65 significant genes fell in this block region (3197 common genes for both NTUH and NCI datasets); while 68 of 88 genes fell in the same block in the NCI dataset. Five genes found in the overlapping region were the common genes among the significant genes from the two datasets, implying these genes were differentially expressed in both Taiwanese and U.K. patients. They are basic transcription factor 3 (BTF3), cyclin-dependent kinase inhibitor 2A (CDKN2A), estrogen receptor 1 (ESR1), GATA binding protein 3 (GATA3), and trefoil factor 3 (TFF3). All of these five genes have already been reported to be associated with ER status in human breast cancers. There were 67 differentially expressed candidate genes appearing exclusively in either the Taiwanese or the U.K. patients, as depicted by the Venn diagram (Fig. 4). These 67 mutually exclusive genes drew our attention since they might reflect the differences between the two populations, and may potentially influence the ER status in each population. Further details about these genes are shown in Table 2.

### Association analysis of SNPs in different ethnic origins

Since the SNP information of many human genes is available, we searched for possible SNP variations of these 67 genes between the representative populations of Taiwanese and U.K. patients in public databases. These results, namely the population-unique SNPs in the mutually exclusive genes, may imply the associations of these SNPs with ER status in breast cancer of different ethnic origins. Perlegen Genotype Browser and dbSNP, two public SNP databases, were useful for our association analysis of SNPs. Perlegen Genotype Browser includes SNP information on three ethnic populations, African American, European American, and Chinese. We chose the SNPs of European American and Chinese to represent the U.K. and Taiwanese populations respectively for further statistical analysis. Using a cutoff p-value of 0.001, Pearson’s chi-square test identified 17 candidate genes which could be potential focuses in breast cancer development, including damage-specific DNA binding protein 2 (DDB2), ATP-binding cassette, sub-family D (ALD) and member 3 (ABCD3), ATPase, Na+/K+ transporting, beta 3 polypeptide (ATP1B3), sphingosine kinase type 1 interacting protein (SKIP), developmentally regulated GTP binding protein 1 (DRG1), interleukin-1 receptor-associated kinase 1 (IRAK1), keratin 7 (KRT7), dipeptidyl-peptidase 6 (DPP6), E2F transcription factor 3 (E2F3), fucosyltransferase 8 (FUT8), hydroxysteroid (17-beta) dehydrogenase 4 (HSD17B4), lipin 1 (LPIN1), myosin VI (MYO6), nuclear factor I/B (NFIB), protein tyrosine phosphatase, non-receptor type substrate 1 (PTPNS1), syndecan 4 (SDC4), and seven in absentia homolog 2 (SIAH2). These genes had more than one statistically significant SNP (p-value < 0.001) in genotype and allele frequency between our and NCI datasets. A total of 83 SNPs among the 17 genes were identified. Genes with the most statistically significant SNPs between European American and Chinese are as follows: DPP6 (19 SNPs), followed by HSD17B4 (13 SNPs), ABCD3 (10 SNPs), and FUT8 (9 SNPs). The remaining genes have fewer than five significant SNPs. In Table 3, the differential genes with their possible related SNPs are presented.

### Functional study of identified genes

In order to understand the functions of the 17 identified genes and their possible relationships with ER expression, we used DAVID database (Database for Annotation, Visualization and Integrated Discovery) (Dennis et al. 2003) to perform this analysis. These genes were functionally classified into the following six categories: alternative splicing (DPP6, PTPNS1, NFIB, MYO6, SKIP, IRAK1, DDB2, and FUT8), signal-anchor (DPP6, ATP1B3, FUT8), sh3-binding (PTPNS1, FUT8), disease mutation (HSD17B4, MYO6, ABCD3, DDB2), peroxisome (HSD17B4, ABCD3), and nucleotide-binding (MYO6, ABCD3, IRAK1, DRG1). As expected, all of the observed functions are either directly or indirectly involved in the regulation of estrogen receptors, in particular the genes closely associated with alternative splicing, sh3-binding, disease mutation, and peroxisome (Bonofiglio et al. 2005; Pfeffer et al. 1996; Troester et al. 2006; Zhou et al. 2006).

## Discussion

According to the result of SNP analysis, 83 SNPs from the 17 differentially expressed genes in either the Taiwanese or U.K. ER+/ER− breast cancers were identified (Table 3). The findings indicate these 17 genes and their related SNPs may be important in the ER regulation in breast cancer of different populations. Since many reports have showed the association of genetic variations of estrogen receptor and breast cancer (Iwase H, 1996; Kang et al. 2002; Roodi et al. 1995), it is worthy to presume that the 83 identified SNPs in ER-related genes may very well have either direct or indirect influence on the ER status in breast cancer of different ethnic populations. Here we used SNPs to represent the polymorphisms between Taiwan and U.K. populations since they are good indicators for measuring the genetic differences between two different ethnic origins. Moreover, among the 17 significant genes mentioned above, only DDB2 was from our data, while the rest of the 16 genes were from the NCI data. DDB2 is known for its function in DNA binding while it also acts as a tumor suppressor. A recent study provides further evidence that rs830083 polymorphisms in DDB2 may contribute to the etiology of lung cancer in Chinese population (Hu et al. 2006). This corresponds to our study in that the SNPs we identified in DDB2 may play a role in specific regulation of estrogen receptor in Chinese breast cancer patients.

Except for DDB2, the expressions of the other 16 genes, ABCD3, ATP1B3, SKIP, DRG1, IRAK1, KRT7, DPP6, E2F3, FUT8, HSD17B4, LPIN1, MYO6, NFIB, PTPNS1, SDC4, and SIAH2, are different in ER-positive and ER-negative U.K. patients. ATP1B3 is derived from the primary differentiation event during mammalian development (Adjaye et al. 2005). IRAK1 has been proposed that one SNP within it, when combined with high-risk genotype at TLR6-1-10, conferred a significant increase in the risk for prostate cancer, suggesting synergistic effects between sequence variants in IRAK1 and the TLR 6-1-10 gene cluster (Sun et al. 2006). LPIN1 is reported to be a candidate gene for human lipodystrophy syndromes as common SNPs in LPIN1 of lipodystrophy patients have been identified (Cao and Hegele, 2002). Limited research has been done on the genetic variants in these genes, but the correlations of these ER-related genes and tumorigenesis are likely to correspond to an increase in susceptibility for breast cancer. Cytokeratin 7 (encoded by KRT7/CK7) is found in the majority of type 1 papillary renal cell carcinomas and chromophobe renal cell carcinomas, and its expression profile alteration is particularly associated with tumorigenesis of primary adenocarcinoma of the small intestine (Chen and Wang, 2004; Mazal et al. 2005). SDC4 is a cell-adhesion molecule related to the enhanced adhesion of cancer cells to fibronectin (Koike et al. 2004), and functions as a receptor in intracellular signaling. A study has showed SKIP to be a protein likely to participate in the regulation of SPHK1 activity modulation, but much about its functions remain unknown. The association between estrogen and SIAH2 has been illustrated by a mechanism in which the estrogen-ER complex markedly reduces the level of N-CoR through a process related to the up-regulation of SIAH2 and the subsequent targeting of N-CoR for proteasomal degradation (Frasor et al. 2005).

The genes, BTG2, ISL1, MCP (also known as CD46), SIAH2, and XBP1, in the list of mutually exclusive subset shown in Table 2 were previously known to be associated with ER status (Frasor et al. 2005; Gay et al. 2000; Kawakubo et al. 2006; Lacroix and Leclercq, 2004; Rushmere et al. 2004). These five ER-regulated genes were only observed to be differentially expressed in the NCI dataset, but not in our dataset. Intriguingly, the majority of published reports used breast cancer cell lines extracted from Caucasian patients in their studies. One possible explanation for this phenomenon is that the association of these five genes and estrogen is solely limited to Caucasian breast cancers patients.

In addition, five genes, BTF3, CDKN2A, ESR1, GATA3, and TFF3, fell in the overlapping subset as shown in Figure 4, meaning they were identified to be differentially expressed in both our and the NCI datasets. All of these genes have been identified and studied to play roles in ER regulation (Doane et al. 2006; Green et al. 2007; Milde-Langosch et al. 2001; Oh et al. 2006). According to a study, specific interaction between BTF3 and ERalpha has been verified *in vivo* and *in vitro*; moreover, BTF3 may influence the mechanism by which the AF-1 (transcriptional activation function) of ERalpha simulates gene expression (Green et al. 2007). GATA3, TFF3, and ESR1 are three estrogen-regulated genes known for their over-expression in luminal subtype A, which is primarily composed of ER+ (Chen and Wang, 2004; Doane et al. 2006). Meanwhile, GATA3 is also a transcription factor serving as a curial component in the tumorigenesis of ER+ breast cancer, and is involved in growth control and maintenance of the differentiated state in epithelial cells (Usary et al. 2004).

The SNPs identified in our selected genes may be involved in determining whether ER expression causes disparities between Chinese and Caucasian breast cancer patients. Our work can provide possible SNPs associated with ER status in breast cancer of different ethnic origins and a set of potential gene expression signatures for novel targeted therapeutic strategies.

## Figures and Tables

**Table 1.** Summary of microarray information and clinical characteristics of NTUH and NCI datase.

**Identifiers**	**NTUH**	**NCI**
Institute	National Taiwan University Hospital	John Radcliffe Hospital
Populations	Taiwanese	Caucasian
Microarray platform	Agilent human 1Av2	cDNA (NCI)
Sample size, No.	59	99
ER status, No. (%)		
Positive	44 (73.3)	65 (65.6)
Negative	15 (25.0)	34 (34.3)
Unknown	0	0
Lymph node status, No. (%)		
Positive	34 (57.6)	53 (53.5)
Negative	21 (35.6)	46 (46.5)
Unknown	4 (6.8)	0
Histological grade, No. (%)		
1	14 (23.7)	16 (16.2)
2	31 (52.5)	38 (38.4)
3	12 (20.3)	45 (45.4)
Unknown	2 (3.3)	0
Tumor size, No. (%)		
≦2 cm	13 (22.0)	36 (36.4)
>2cm	20 (33.9)	63 (63.6)
Unknown	26 (44.1)	0
Reference	this study	Sotiriou et al. [9]

* ER: estrogen receptor.

**Table 2.** Subsets of differentially expressed genes in NTUH and NCI datasets.

**Gene symbol**	**Gene name**	**p-value^‡^**	**q-value^‡^**	**source^§^**	**ER+/ER−**
Intersection subset					
BTF3	Basic transcription factor 3	1.10E-06	0.000189	T	↑
		1.35E-06	3.43E-05	U	↑
TFF3	Trefoil factor 3 Cyclin-dependent	5.48E-07	0.000135	T	↑
		1.35E-06	3.43E-05	U	↑
ESR1*	Estrogen receptor 1	5.48E-07	0.000135	T	↑
		1.35E-06	3.43E-05	U	↑
		1.35E-06	3.43E-05		
GATA3*	GATA binding protein 3	5.48E-07	0.000135	T	↑
		1.35E-06	3.43E-05	U	↑
		1.35E-06	3.43E-05		
CDKN2A*	kinase inhibitor 2A	5.48E-07	0.000135	T	↓
		1.35E-06	3.43E-05	U	↓
		1.35E-06	3.43E-05		
Mutually exclusive subset					
DDB2	Damage-specific DNA binding protein 2	5.48E-07	0.000135	T	↑
KIAA0020	KIAA0020	5.48E-07	0.000135	T	↓
NPY1R	Neuropeptide Y receptor Y1	5.48E-07	0.000135	T	↑
TCEAL1	Transcription elongation factor A (SII)-like 1	1.64E-06	0.000247	T	↑
ABCD3	ATP-binding cassette, sub-family D (ALD), member 3	1.35E-06	3.43E-05	U	↑
AOC3	Amine oxidase, copper containing 3 (vascular adhesion protein 1)	1.35E-06	3.43E-05	U	↑
APS	Adaptor protein with pleckstrin homology and src homology 2 domains	1.35E-06	3.43E-05	U	↓
ATP1B3	ATPase, Na+/K+ transporting, beta 3 polypeptide	1.35E-06	3.43E-05	U	↓
BLR1	Burkitt lymphoma receptor 1, GTP binding protein (chemokine (C-X-C motif) receptor 5)	2.70E-06	5.46E-05	U	↓
BTG2	BTG family, member 2	1.35E-06	3.43E-05	U	↑
BUB1	BUB1 budding uninhibited by benzimidazoles 1 homolog (yeast)	1.35E-06	3.43E-05	U	↓
CCNE1	Cyclin E1	1.35E-06	3.43E-05	U	↓
CCNG2*	Cyclin G2	1.35E-06	3.43E-05	U	↑
CD79A*	CD79a molecule	1.35E-06	3.43E-05	U	↓
		2.70E-06	5.46E-05		
CDK2AP1*	CDK2-associated protein 1	1.35E-06	3.43E-05	U	↓
		1.35E-06	3.43E-05		
CFLAR*	CASP8 and FADD-like apoptosis regulator	1.35E-06	3.43E-05	U	↓
		1.35E-06	3.43E-05		
DMWD	dystrophia myotonica-containing WD repeat motif	2.70E-06	5.46E-05	U	↓
DPP6	Dipeptidyl-peptidase 6	1.35E-06	3.43E-05	U	↑
DRG1	Developmentally regulated GTP binding protein 1	2.70E-06	5.46E-05	U	↓
DSC2	Desmocollin 2	2.70E-06	5.46E-05	U	↓
E2F3*	E2F transcription factor 3	1.35E-06	3.43E-05	U	↓
		1.35E-06	3.43E-05		
FABP7	Fatty acid binding protein 7, brain	1.35E-06	3.43E-05	U	↓
FCGRT	Fc fragment of IgG, receptor, transporter, alpha	1.35E-06	3.43E-05	U	↑
FUT8	Fucosyltransferase 8 (alpha (1,6) fucosyltransferase)	1.35E-06	3.43E-05	U	↑
GATA6	GATA binding protein 6	1.35E-06	3.43E-05	U	↓
GBP1	Guanylate binding protein 1, interferon-inducible, 67 kDa	1.35E-06	3.43E-05	U	↓
HSD17B4	Hydroxysteroid (17-beta) dehydrogenase 4	1.35E-06	3.43E-05	U	↑
HSPC121	protein tyrosine phosphatase-like A domain containing 1	1.35E-06	3.43E-05	U	↑
IL2RG	Interleukin 2 receptor, gamma (severe combined immunodeficiency)	2.70E-06	5.46E-05	U	↓
IL6ST	Interleukin 6 signal transducer (gp130, oncostatin M receptor)	1.35E-06	3.43E-05	U	↑
ILK	Integrin-linked kinase	1.35E-06	3.43E-05	U	↓
IMPDH1	IMP (inosine monophosphate) dehydrogenase 1	1.35E-06	3.43E-05	U	↓
IRAK1	Interleukin-1 receptor-associated kinase 1	1.35E-06	3.43E-05	U	↓
ISL1	ISL1 transcription factor, LIM/homeodomain, (islet-1)	1.35E-06	3.43E-05	U	↑
KLK5	Kallikrein 5	1.35E-06	3.43E-05	U	↓
KRT6B*	keratin 6B	1.35E-06	3.43E-05	U	↓
		1.35E-06	3.43E-05		
KRT7	keratin 7	1.35E-06	3.43E-05	U	↓
LPIN1	Lipin 1	1.35E-06	3.43E-05	U	↓
LRBA	LPS-responsive vesicle trafficking, beach and anchor containing	1.35E-06	3.43E-05	U	↑
LYN*	V-yes-1 Yamaguchi sarcoma viral related oncogene homolog	1.35E-06	3.43E-05	U	↓
		1.35E-06	3.43E-05		
MAP2K3	Mitogen-activated protein kinase kinase 3	1.35E-06	3.43E-05	U	↓
MCM6	MCM6 minichromosome maintenance deficient 6 (MIS5 homolog, S. pombe) (S. cerevisiae)	1.35E-06	3.43E-05	U	↓
MCP(CD46)	membrane cofactor protein	1.35E-06	3.43E-05	U	↑
MMP7	Matrix metallopeptidase 7 (matrilysin, uterine)	1.35E-06	3.43E-05	U	↓
MSN	Moesin	1.35E-06	3.43E-05	U	↓
MYB*	V-myb myeloblastosis viral oncogene homolog (avian)	1.35E-06	3.43E-05	U	↑
		1.35E-06	3.43E-05		
MYBL1	v-myb myeloblastosis viral oncogene homolog (avian)-like 1	1.35E-06	3.43E-05	U	↓
MYC	V-myc myelocytomatosis viral oncogene homolog (avian)	8.09E-06	0.000104	U	↑
MYO6	myosin VI	1.35E-06	3.43E-05	U	↑
NFATC3	Nuclear factor of activated T-cells, cytoplasmic, calcineurin-dependent 3	1.35E-06	3.43E-05	U	↓
NFIB	Nuclear factor I/B	1.35E-06	3.43E-05	U	↓
NME3*	Non-metastatic cells 3, protein expressed in	1.35E-06	3.43E-05	U	↑
		1.35E-06	3.43E-05		
NP	Nucleoside phosphorylase	1.35E-06	3.43E-05	U	↓
NSEP1	Y box binding protein 1	1.35E-06	3.43E-05	U	↓
NUMA1	Nuclear mitotic apparatus protein 1	1.35E-06	3.43E-05	U	↑
OAS1	2′,5′-oligoadenylate synthetase 1, 40/46 kDa	1.35E-06	3.43E-05	U	↑
PFKP	Phosphofructokinase, platelet	1.35E-06	3.43E-05	U	↓
PTP4A2*	Protein tyrosine phosphatase type IVA, member 2	1.35E-06	3.43E-05	U	↑
		1.35E-06	3.43E-05		
PTPNS1	protein tyrosine phosphatase, non-receptor type substrate 1	1.35E-06	3.43E-05	U	↓
RAB7	RAB7, member RAS oncogene family	1.35E-06	3.43E-05	U	↑
SDC4	Syndecan 4 (amphiglycan, ryudocan)	1.35E-06	3.43E-05	U	↑
SIAH2*	Seven in absentia homolog 2 (Drosophila)	1.35E-06	3.43E-05	U	↑
		1.35E-06	3.43E-05		
		1.35E-06	3.43E-05		
SKIP	SPHK1 (sphingosine kinase type 1) interacting protein	1.35E-06	3.43E-05	U	↑
UCP2	Uncoupling protein 2 (mitochondrial, proton carrier)	2.70E-06	5.46E-05	U	↓
VAMP2	Vesicle-associated membrane protein 2 (synaptobrevin 2)	1.35E-06	3.43E-05	U	↓
WWP1	WW domain containing E3 ubiquitin protein ligase 1	1.35E-06	3.43E-05	U	↑
XBP1	X-box binding protein 1	1.35E-06	3.43E-05	U	↑

* The expression values of these genes were detected by two or more probes in the microarrays from either NTUH or NCI dataset. Hence two or more p-values and q-values were calculated and shown together.

‡ p-value and q-value were calculated using the software, EDGE. Numbers have been rounded off.

§ T for NTUH dataset and U for NCI dataset.

↑, higher expression in ER+ samples; ↓lower expression in ER- samples.

**Table 3.** List of identified genes and SNPs.

**ER+/ER−**	**Gene symbol**	**SNP ID**	Dominant genotype*	**p-value**	**Dominant**	**p-value**	**odd ratios**
↑	DDB2	rs3781619^b^	GG/AA	1.09E-05	G/A	6.21E-08	0.05 (7.9E-3∼0.17)
		rs2306353^c^	TT/AA	1.09E-05	T/A	6.21E-08	0.05 (7.9E-3∼0.17)
		rs2050648^b^	AG/GG	4.45E-04	A/G	5.02E-05	7.36 (2.59–23.50)
		rs10493872^f^	GT/TT	6.14E-06	T/T	1.80E-06	Inf (7.52-Inf)
		rs4847303^f^	AG/GG	4.45E-04	A/G	5.02E-05	7.36 (2.59–23.50)
		rs12143221^b^	AG/AA	4.45E-04	G/A	5.02E-05	0.14 (0.04–0.39)
↑	ABCD3	rs2147794^b^	AG/AA	4.45E-04	G/A	5.29E-05	0.13 (0.04–0.39)
		rs17410399^b^	AT/AA	4.45E-04	T/A	5.29E-05	0.13 (0.04–0.39)
		rs1158254^a^	AC/CC	4.45E-04	A/C	5.02E-05	7.36 (2.59–23.50)
		rs16946^b^	AG/GG	4.45E-04	A/G	5.02E-05	7.36 (2.59–23.50)
		rs4148050^b^	AG/GG	4.45E-04	A/G	5.02E-05	7.36 (2.59–23.50)
		rs2296382^e^	CT/CC	4.45E-04	T/C	5.02E-05	0.14 (0.04–0.39)
↓	ATP1B3	rs2068229^b^	AA/GG	7.56E-04	A/G	3.68E-05	6.77 (2.56–19.32)
↑	SKIP	rs1879488^a^	CC/AC	5.57E-07	C/C	7.09E-05	0 (0–0.12)
		rs2273252^d^	CC/CG	6.14E-06	C/C	4.00E-06	Inf (6.89-Inf)
↓	DRG1	rs6518745^e^	TT/CC	1.98E-07	T/C	3.70E-11	0.01 (2.3E-4∼0.07)
		rs5994397^d^	CC/GG	1.91E-07	C/G	1.16E-11	107.32 (15.37–4591.03)
↓	IRAK1	rs1059703^b^	AA/GG	1.37E-04	A/G	3.34E-07	10.95 (3.99–32.85)
		rs7061789^b^	AA/GG	9.85E-06	A/G	9.10E-09	16.88 (5.68–57.34)
		rs936331^e^	CC/TT	1.69E-04	C/T	1.38E-05	0.13 (0.04–0.35)
↓	KRT7	rs1317649^e^	CC/TT	1.69E-04	C/T	1.38E-05	0.13 (0.04–0.35)
		rs1870220^b,b^	GG/AA	1.69E-04	G/A	1.38E-05	0.13 (0.04–0.35)
		rs878742^e^	CC/TC	7.00E-05	C/C	9.45E-05	0.11 (0.02–0.37)
		rs4960626^b^	AA/AG,GG	1.00E-04	A/G	2.37E-06	14.79 (4.12–68.87)
		rs4960625^b^	AA/AG	3.98E-05	A/G	1.44E-05	11.78 (3.37–53.66)
		rs1568900^f^	GG/GT	2.13E-05	G/T	2.27E-06	11.69 (3.74–44.64)
		rs877471^e^	CC/CT	1.70E-05	C/C,T	5.62E-06	14.57 (3.85–83.17)
		rs1016103^d^	CC/CG	1.35E-05	C/G	1.57E-06	13.71 (4.07–60.83)
		rs877472^e^	TT/CT	1.70E-05	T/C,T	5.62E-06	0.07 (0.01–0.26)
		rs940848^b^	AA/AG	2.13E-05	A/G	2.27E-06	11.69 (3.74–44.64)
		rs3800573^e^	TT/CT	2.23E-05	T/C	5.45E-06	0.09 (0.02–0.29)
↑	DPP6	rs3800574^c^	AA/AT	3.44E-05	A/T	5.80E-06	10.86 (3.45–41.68)
		rs868880^b^	AA/AG	3.34E-05	A/G	2.27E-06	0.09 (0.02–0.27)
		rs3778735^b^	GG/AG	8.97E-06	G/A	1.18E-06	0.07 (0.02–0.24)
		rs3778739^b^	AA/AG	5.27E-05	A/G	7.66E-06	10.60 (3.37–40.71)
		rs3778740^a^	CC/AC	5.49E-05	C/A	6.51E-06	0.08 (0.02–0.27)
		rs2293356^e^	CC/TC	3.92E-04	C/T	1.93E-05	0.10 (0.03–0.32)
		rs2293355^d^	CC/GC	4.92E-04	C/G	9.70E-06	0.10 (0.03–0.31)
		rs2293354^b^	GG/AG	4.92E-04	G/A	9.70E-06	0.10 (0.03–0.31)
		rs17515800^b^	GG/GG	3.36E-04	G/G	1.88E-05	Inf (5.73-Inf)
		rs1110077^f^	TT/GT	6.68E-05	T/G	7.41E-06	0.10 (0.03–0.31)
		rs9348428^e^	CC/CT	1.30E-05	C/T	3.96E-06	9.49 (3.33–30.65)
↓	E2F3	rs9348429^e^	TT/CC	6.68E-05	T/C	7.41E-06	0.10 (0.03–0.31)
		rs16883824^e^	CC/CT	8.35E-05	C/T	2.23E-05	8.00 (2.82–25.63)
		rs17826580^a^	AA/AC	5.47E-04	A/A	2.81E-04	7.76 (2.50–29.34)
		rs8018909^d^	GG/CG	5.47E-04	G/G	2.81E-04	0.13 (0.03–0.40)
		rs17246259^b^	GG/AG	4.01E-04	G/A	6.31E-05	0.12 (0.03–0.37)
		rs17826736^e^	TT/CT	4.01E-04	T/C,T	6.31E-05	0.12 (0.03–0.37)
↑	FUT8	rs2268957^e^	CC/CT	5.47E-04	C/C	2.81E-04	7.76 (2.50–29.34)
		rs17826820^b^	GG/AG	5.47E-04	G/G	2.81E-04	0.13 (0.03–0.40)
		rs1998035^b^	AA/AG	5.47E-04	A/A	2.81E-04	7.76 (2.50–29.34)
		rs2300871^a^	AA/AC	5.47E-04	A/A	2.81E-04	7.76 (2.50–29.34)
		rs2268960^b^	AA/AG	4.01E-04	A/A,G	6.31E-05	8.40 (2.69–31.91)
		rs26181^e^	CT/TT	1.56E-05	T/T	7.52E-06	18.89 (4.12–178.26)
		rs32651^b^	AG/AA	4.32E-06	A,G/A	1.41E-06	0.04 (4.8E-3∼0.21)
		rs463513^b^	AG/GG	4.95E-05	G/G	1.68E-05	0.06 (6.0E-3∼0.26)
		rs2459726^c^	AT/TT	1.91E-04	T/T	1.30E-04	10.46 (2.74–59.95)
		rs2678070^b^	AG/GG	8.22E-05	G/G	7.81E-05	14.68 (3.17–139.58)
↑	HSD17B4	rs382719^e^	CT/CC	1.60E-05	C/C	1.68E-05	17.39 (3.78–164.30)
		rs2636968^b^	AG/AA	1.60E-05	A/A	1.68E-05	0.06 (6.0E-3∼0.26)
		rs26184^e^	CT/CC	1.60E-05	C/C	1.68E-05	0.06 (6.0E-3∼0.26)
		rs2636961^e^	CT/TT	6.34E-05	C/T	6.15E-05	18.64 (4.93–106.85)
		rs2678074^f^	GT/TT	6.34E-05	G/T	6.15E-05	18.64 (4.93–106.85)
		rs2636962^a^	AC/CC	6.34E-05	A/C	6.15E-05	18.64 (4.93–106.85)
↓	LPIN1	rs4129757^b^	GG/AA	7.97E-05	G/A	2.35E-06	10.38 (3.58–33.54)
		rs2748956^d^	CC/CG	5.47E-04	C/C	2.81E-04	7.12 (2.27–27.08)
		rs1280050^b^	AG/GG	5.12E-05	G/G	3.66E-05	15.99 (3.47–151.33)
↑	MYO6	rs1280054^b^	AG/GG	5.12E-05	G/G	3.66E-05	15.99 (3.47–151.33)
		rs910679^e^	CT/CC	5.12E-05	C/C	3.66E-05	0.06 (6.0E-3∼0.26)
		rs1280053^b^	AG/AA	5.12E-05	A/A	3.66E-05	0.06 (6.0E-3∼0.26)
		rs12236761^c^	TT/AT	5.57E-07	T/T	1.44E-07	0 (0–0.10)
↓	NFIB	rs10961439^f^	TT/GT	1.68E-07	T/G	2.28E-08	0.06 (0.02–0.19)
		rs12684749^b^	AA/AG	3.59E-08	A/A,G	2.46E-04	Inf (4.40-Inf)
		rs10810120^e^	TT/CT	2.00E-07	T/C	6.55E-05	0.07 (0.02–0.20)
		rs2267905^a^	AA/AC	3.70E-08	A/C	5.05E-09	36.71 (7.99–348.43)
↓	PTPNS1	rs2267906^e^	TT/CT	1.05E-06	T/C	9.57E-08	0.04 (3.7E-3∼0.16)
		rs3197744^f^	TT/GG	5.17E-06	T/G	9.73E-10	0.03 (5.5E-3∼0.12)
↑	SDC4	rs6073718^b^	AG/AA	3.46E-05	A/A	1.94E-05	0.03 (7.7E-4∼0.23)
		rs2267868^e^	CT/TT	3.46E-05	T/T	1.94E-05	29.89 (4.31–1297.07)
		rs6789306^b^	AA/AG	5.55E-07	A/G	8.85E-07	43.10 (6.17–1873.77)
↑	SIAH2	rs7615292^b^	GG/AG	3.83E-06	G/G	9.69E-06	0.03 (6.9E-4∼0.21)
		rs16862837^f^	GG/GT	4.45E-06	G/G	3.30E-06	20.52 (4.48–193.32)
		rs1879421^b^	GG/AG	1.62E-06	G/G	5.33E-06	0 (0–0.14)

a Genotype frequency: AA, AC, CC. Allele frequency: A, C.

b Genotype frequency: AA, AG, GG. Allele frequency: A, G.

c Genotype frequency: AA, AT, TT. Allele frequency: A, T.

d Genotype frequency: CC, CG, GG. Allele frequency: C, G.

e Genotype frequency: CC, CT, TT. Allele frequency: C, T.

f Genotype frequency: GG, GT, TT. Allele frequency: G, T.

**Note:** Dominant genotype and allele represent the groups with the highest genotype and allele frequency. The left parts of the genotype and allele represent Caucasian, whereas the right parts represent Chinese.

↑, higher expression in ER+ samples; ↓ lower expression in ER- samples.
